# Nectin4 is a potential therapeutic target for asthma

**DOI:** 10.3389/fimmu.2022.1049900

**Published:** 2022-11-15

**Authors:** Pureun-Haneul Lee, Seon Muk Choi, Min Hyeok An, Da Yeon Hwang, Shinhee Park, Ae Rin Baek, An-Soo Jang

**Affiliations:** ^1^ Department of Interdisciplinary Program in Biomedical Science Major, Graduate School of Soonchunhyang University, Soonchunhyang University Bucheon Hospital, Bucheon, South Korea; ^2^ Division of Allergy and Respiratory Medicine, Department of Internal Medicine, Soonchunhyang University Bucheon Hospital, Bucheon-si, Gyeonggi-do, South Korea

**Keywords:** adherens junction protein, asthma, epithelial barrier, Nectin4, therapeutic target

## Abstract

**Background:**

Nectins comprise a family of cellular adhesion molecules involved in Ca^2+^-independent cellular adhesion. Neither the biological significance nor clinical potential of Nectin4 for asthma has been investigated.

**Objectives:**

The aims of this study were to elucidate the role of Nectin4 in airway inflammation and to determine the relationship between Nectin4 and clinical variables in patients with asthma.

**Methods:**

The relationship between Nectin4 levels in the blood of asthmatic patients and clinical variables was examined. *Dermatophagoides pteronyssinus* 1 (*Der p1*)-exposed normal human bronchial epithelial (NHBE) cells, and Nectin4-deficient (Nectin4^−/−^) and wild-type (WT) mice sensitized/challenged with ovalbumin (OVA), were used to investigate the involvement of Nectin4 in the pathogenesis of bronchial asthma *via* the Src/Rac1 pathway.

**Results:**

Plasma Nectin4 levels were significantly higher in asthmatic patients than controls and correlated with specific IgE D1, D2, lung function. The ROC curves for Nectin4 levels differed between asthma patients and controls. Nectin4/Afadin and Src/Rac1 levels were significantly increased in NHBE cells exposed to *Der* p1, but decreased in NHBE cells treated with Nectin4 siRNA. Airway obstruction and inflammation, as well as the levels of Th2 cytokines, Nectin4, and Src/Rac1, were increased in WT OVA/OVA mice compared with WT sham mice. Nectin4 knockdown resulted in lower levels of Afadin and Src/Rac1 in Nectin4^−/−^OVA/OVA than WT OVA/OVA mice.

**Conclusion:**

These results suggest that Nectin4 is involved in airway inflammation and may be a therapeutic target in patients with asthma.

## Introduction

The bronchial epithelium maintains lung tissue homeostasis and protects the lung against pathogens (1, 2). Recent studies have emphasized the importance of epithelial barrier-derived cytokines for the activation of Th2 immune responses and interactions between Th2 effectors and other immune system components (1–3). Disruption of the regulatory functions of the airway epithelial barrier triggers allergic airway inflammation and thus asthma development (3).

Nectins (Nectin-1 to -4) are immunoglobulin superfamily members containing three Ig-like loops (V and two C2-type domains) that act as a PDZ‐binding domain (4). Nectins are ubiquitously expressed and, together with other junctional proteins, promote intercellular junction formation (5). Nectins can be found in the adherens junctions (AJs) of polarized epithelial cells, at neuronal synapses, and at points of contact between cultured epithelial cells (6). Nectins bind to Afadin through its cytoplasmic tail and associate with the actin cytoskeleton. Afadin acts as an adaptor protein by further binding scaffolding and F-actin-binding proteins, and contributes to the association of Nectins with several other cell-cell adhesion and intracellular signaling proteins, such as those of the PI3k-Akt pathway (7–9), either in collaboration with or independent from cadherins (10, 11). Nectins are also novel regulators of cellular activities, including cell polarization, differentiation, movement, and proliferation, and they also support cell survival (11). Nectin4 contributes to cell growth, angiogenesis, and the proliferation of human lung adenocarcinoma cells through the Rac1-signaling pathway; it has therefore been linked to a poor prognosis in lung cancer patients (5, 11, 12). Together with other Nectins and Necls, Nectin4 plays an important role in both acquired immunity and angiogenesis, suggesting a much broader range of functions for this protein (13, 14). In Nectin-deficient transgenic mice, abnormal formations of ectodermal tissues, including in the eye, tooth, inner ear, and skin, are observed (15–17).

The role of Nectin4 in asthma is not fully understood. In this study, we investigated whether the absence of Nectin4 contributes to the pathogenesis of asthma, and the signaling pathways involved in this process. We also investigated the relationship between clinical variables and plasma Nectin4 levels in patients with asthma.

## Materials and methods

### Subjects

All subjects had a clinical diagnosis of asthma according to Global Initiative for Asthma (GINA) guidelines (18) that was supported by one or more of the following criteria: 1) variability in the maximum diurnal peak expiratory flow of >20% over the course of 14 days, 2) an increase in FEV1 of >15% after inhalation of 200-400 μg albuterol, or 3) a 20% reduction in FEV1 in response to a provocative concentration of inhaled methacholine (PC20 methacholine) of less than 10 mg/ml. All subjects underwent standardized assessments that included complete blood cell and differential counts, IgE measurement, chest posteroanterior radiography, allergy skin prick tests, and spirometry. Asthma exacerbation was defined as episodes of progressive increase in shortness of breath, cough, wheezing, or chest tightness, or some combination of these symptoms, accompanied by decreases in expiratory airflow and use of systemic corticosteroids (tablets, suspension, or injection), or an increase from a stable maintenance dose, for at least 3 days, and a hospitalization or emergency department visit because of asthma, requiring systemic corticosteroids. Normal control subjects were recruited from spouses of the subjects or members of the general population who answered negatively to a screening questionnaire regarding respiratory symptoms and other allergic diseases, had FEV1 values over 80% predicted, PC20 methacholine over 10 mg/ml, and normal findings on chest radiographs. The biospecimens and clinical data were provided by the biobank of Soonchunhyang University Bucheon Hospital, a member of the Korea Biobank Network. This study was approved by the Soonchunhyang University Bucheon Hospital Institutional Review Board (SCHBC 2020-05-038-003).

### Cell culture

Primary normal human bronchial epithelial (NHBE) cells (CC-2540, Lonza, Walkersville, MD) (3500cells/cm^2^) were maintained as previously described (19). Cells were placed in bronchial epithelial cell growth medium (BEGM, CC-3170, Lonza) without supplements for 24 h and then stimulated with 10μg/ml *Dermatophagoides pteronyssinus* 1 (*Der* p1, Arthropods of Medical Importance Resource Bank, Institute of Tropical Medicine, Yonsei University). In separated tests, NHBE were transfected with small interfering RNA (siRNA) duplexes designed against Nectin4 *or* nonspecific siRNA control (1027418, Qiagen, CA, USA). NHBE cells cultured in 6-well plates were transfected with 100 nM siRNA or negative control using Lipofectamine 2000 (11668019, Invitrogen, CA, USA). After 24hr, cells were treated with 10 μg/ml Der p1 and harvested for western blotting. Trans-epithelial electrical resistance (TEER) was measured using an Epithelial Volt/Ohm Meter (EVOM) (EVOM2, World Precision Instruments, FL). TEER values are expressed by raw oh m values minus the oh m value of a blank insert, multiplied by the area of the insert (1.12 cm^2^).

### Animals

All experimental animal methods followed a protocol approved by the Institutional Animal Care and Use Committee of the Soonchunhyang University Bucheon Hospital (SCHBC-animal-2020-11). Female 6-week-old BALB/c mice (n=6-10 mice per group) were sensitized by means of intraperitoneal injection at days 0 and 14 with 50 mg of grade V chicken egg OVA (A5503, Sigma-Aldrich, St Louis, Mo) that was emulsified in 10 mg of hydroxyl aluminum plus 100 mL of Dulbecco PBS. At days 21 to 23, all mice received intranasal challenges with 150 mg of grade III OVA (5378, Sigma-Aldrich) in 50 mL of Dulbecco PBS. Control mice were sensitized and challenged with saline. Airway hyperresponsiveness (AHR) was measured, bronchoalveolar lavage fluid (BALF) was collected, and lung tissue was processed for protein, RNA, and hematoxylin and eosin (H&E), periodic acid Schiff (PAS), Masson trichrome stain, and confocal imaging.

### CRISPR/Cas9 for Nectin4 gene knockout

We purchased SpCas9 protein and sgRNAs to generate Nectin4 knockout mice from Macrogen, Inc. (Seoul, Korea). To knock out the Nectin4 gene (NC_000067.6), we designed six sgRNAs (1 (intron1-2-gR1), AGTGCA AGAGTAGCCCCAGA; 2 (intron1-2-gR2), AGGGGGACAGTCAGAACCAATGG; 3 (intron1-2-gR3), GCAAAGGCGGCCGGAACTTCTGG; 4 (intron7-8-gR1), GTGCTAAGGTTGGGTGCATGGGG; 5 (intron7-8-gR2), AGGTTGGGTGCATGGGGTCGGGG; 6 (intron7-8-gR3), TCGGGGGGACATGGGCACACAGG) and its 5′ upstream sequences of the Nectin4 gene and validated them using the T7E1 *in vitro* cleavage reaction of template DNAs, which were amplified by PCR (F1:5′-TGTCCTTGGTTTCCTGGTTC-3′ and R1: 5′-GGTTCACATGAAGCCCGTAT-3′, F2: 5′-GCATCACCTACCATGCACAC-3′ and R2: 5′-AGTGCAAGAGTAGCCCCAGA-3′). Briefly, the amplified template DNA was incubated for 90 min at 37°C with Cas9 protein (20 nM) and sgRNA (40 nM) in 1× NEB 3 buffer. Reactions were stopped with 6× stop solution containing 30% glycerol, 1.2% SDS, and 100 mM EDTA. Cleavage activity was confirmed by electrophoresis of the reaction mixture.

### Generation of Nectin4 gene knockout mice

Nectin4 knockout mice were generated by Macrogen, Inc., and were interbred and maintained in a pathogen-free condition at Macrogen, Inc. (Seoul, Korea). All manipulations were conducted with the Institutional Animal Care and Use Committee approval. Briefly, pregnant mare serum gonadotropin (PMSG) and human chorionic gonadotropin (hCG) were injected into C57BL/6N female mice. After 48 h, these female mice were mated with C57BL/6N stud male mice. Next day, virginal plug-checked female mice were sacrificed and fertilized embryos were harvested. The mixture of sgRNA and SpCas9 protein was microinjected into one-cell embryos, and microinjected embryos were incubated at 37°C for 1–2 h. Then, 14 to 16 injected one-cell-stage embryos were transplanted into oviducts of pseudopregnant recipient mice (ICR). After F0 mice were born, genotyping was done by direct PCR and sequencing methods using tail-cut samples (Forward primer, 5′- TGTCCTTGGTTTCCTGGTTC-3′; and Reverse primer, 5′- AGTGCAAGAGTAGCCCCAGA-3′). Among of the founders with altered sequences, we selected F0 mice with deletion of exon2&7 sequences of Nectin4.

### Genotyping

Genomic DNA was isolated from the lung tissues of each mouse using a QIAamp DNA mini Kit (51304, Qiagen, MD, USA). Genotyping was done by PCR amplification using the following primers: Forward primer, 5′- TGTCCTTGGTTTCCTGGTTC-3′; and Reverse primer, 5′- AGTGCAAGAGTAGCCCCAGA-3′. The PCR products were visualized by agarose gel electrophoresis; a 6203 bp fragment was amplified in the wild-type mice, and a 443 bp fragment in Nectin4-null mice.

### ELISA

Protein levels of Nectin4 (MBS167116, Mybiosource, CA, USA) in human plasma and IL-4 (M4000B, R&D System, MN, USA), IL-5 (M5000, R&D System), TNF-α (MTA00B, R&D System), IFN-γ (MIF00, R&D System) in mouse BALF and lung proteins were measured by ELISA. A low detection limits were set at 6.56pg/ml for Nectin4, respectively on the basis of the manufacturer’s recommendation.

### Histological analysis

All stains were performed in accordance with the manufacturer’s protocol. The degree of inflammatory cell infiltration in the airway stained with H&E was scored in a double-blind manner by 2 independent observers. The peribronchiolar/perivascular inflammation scores for each view-field were determined as follows: 0, normal; 1, few cells; 2, a ring of inflammatory cells 1 cell layer deep; 3, a ring of inflammatory cells 2–4 cells deep; 4, a ring of inflammatory cells of >4 cells deep. The PAS-positive goblet cells were counted manually, normalized against the length of the bronchial epithelial perimeter on the basal side, and expressed as the number of PAS-positive cells per millimeter of basement membrane. To evaluate collagen deposition of lungs, the area stained with Masson’s trichrome in each section was semi-quantitatively calculated using the ImageJ program (National Institutes of Health, Bethesda, MD).

### Western blot

Protein extracts of mouse lung tissue were collected as previously described (19). Protein was separated by SDS-PAGE and transferred to polyvinylidene fluoride (PVDF) membranes. The membranes were blocked for 5% bovine serum albumin (BSA) in 0.1% Tween 20 in Tris-buffered saline (TBS) (21°C, 2h) and incubated with anti- Nectin4, Afadin, p-Src, Src, Rac1-GTP, Rac1 (4°C, overnight) followed by horseradish peroxidase (HRP)-conjugated secondary antibodies. Detection was performed using WEST-ZOL plus Western Blot Detection System (16024, iNtRon, SungNam, Korea). The relative abundance of protein was determined by quantitative densitometry data were normalized to actin, beta (A2228, Sigma-Aldrich, MA, United States).

### Immunohistochemistry

Mouse lung sections were de-paraffinized and rehydrated in an ethanol series. The sections were treated with 1.4% H_2_O_2_ in methanol for 30 min to inhibit endogenous peroxidase, and then treated for non-specific binding with 1.5% horse serum and incubated with the anti- Nectin4, Afadin, p-Src, Src, Rac1-GTP, Rac1. The next day, sections were incubated with avidin and biotinylated horseradish peroxidase macromolecular complex (PK-4001, Vector Laboratories, Burlingame, CA). Color reaction was developed by staining with liquid DAB+ substrate kit (C09-100, Golden Bridge International Inc., Mukilteo, WA). After immunohistochemical staining the slides were counterstaining the slide with Gill’s hematoxylin for 1 min. Images were analyzed with the ImageJ program (National Institutes of Health, Bethesda, MD).

### Immunofluorescence imaging

Staining was performed on mouse lung sections. Samples were blocked for non-specific binding with 1.5% horse serum and incubation overnight at 4°C with Nectin4 and Rac1-GTP followed by Donkey polyclonal anti-Rabbit IgG H&L (Alexa Fluor 488) (ab150073, Abcam, Cambridge, MA) and Donkey Anti-Mouse IgG H&L (PE) (ab7003, Abcam, Cambridge, MA). Nuclei were counterstained with 4’,6-diamidino-2-phenylindole (DAPI) (Ab104139, Abcam, Cambridge, MA). Sections were observed using confocal laser scanning microscopy (LSM 510 META) and images were generated using the Zeiss LSM image browser (Carl Zeiss Microsystems, Thornwood, NY). Quantification of the immunofluorescent staining was performed with the ImageJ software (NIH, Bethesda, MD). Nectin4 and Rac1-GTP immunostained structures were detected by thresholding technique after subtraction of background. The area of immunostaining for Nectin4 and Rac1-GTP in corresponding nucleus was related to the area of interest (at least 6 sections) and expressed as the mean of relative area (%) ± SD.

### Statistical analysis

Data were presented as means ± standard error of the mean (SEM) or median (range) using SPSS version 22 (SPSS, Chicago, IL). Comparisons of nonparametric variables and parametric variables were performed using Kruskal-Wallis tests and ANOVA respectively, and then *post hoc* analyses using Dunn-tests or turkey-HSD test were performed. Correlations between outcome measures were evaluated by calculating Pearson or Spearman correlation coefficients analysis. A Values of *P*<0.05 were deemed to indicate statistical significance.

## Results

### Increased Nectin4 plasma levels in asthma patients

Sixty-two patients with asthma (duration: 0.21 ± 0.74 or 1.62 ± 3.28 years) and 60 controls were recruited (**Table 1**). The initial FEV1% pred., FVC% pred., FEV1/FVC%, and methacholine PC20 values were significantly lower, and the total IgE and blood eosinophil levels were significantly higher in the asthma patients than in the controls. There was no significant difference in the body mass indices between the two groups.

**Table 1:** Clinical characteristics in control subjects and patients with asthma.

		Control subjects	Asthma Patients	
			Steroid-N	Steroid-Y
subjects (N)		60	51	11
Sex (M/F)		19 / 41	18 / 33	3 / 8
Age (range)		51 (41-77)	46 (30-72)	37 (32-83)
Asthma period, year		-	0.21 ± 0.74	1.62 ± 3.28^#^
Smoke (NS/ES/SM)		56 / 1 / 3	43 / 2 / 6	7 / 0 / 4
Lung function
	FEV1, %pred.	92.40 ± 20.43	79.89 ± 12.15	71.91 ± 19.95^*^
	FVC, %pred.	84.30 ± 15.07	77.73 ± 17.23^*^	72.18 ± 15.73^*^
	FEV1/FVC, %	84.71 ± 5.04	76.62 ± 8.97^*^	75.54 ± 11.08^*^
BMI		24.50 ± 3.56	24.45 ± 3.06	24.45 ± 3.06
PC20, mg/ml		–	7.45 ± 8.40^*^	11.41 ± 10.48^*^
Total _IgE, IU/mL		91.83 ± 314.19	191.20 ± 344.73^*^	129.26 ± 254.07
Skin_test_positive, %		7 (11.6%)	19 (37.3%)	2 (18.2%)
Blood_WBC, uL		6583.82 ± 2299.1	7021.33 ± 2260.04	8143.00± 2970.20
Blood Eosinophil, %		2.69 ± 2.67	5.05 ± 5.49^*^	4.45 ± 3.54

Data expressed as mean ± SD or median (range); SM; smoker, ES; ex-smoker, NS; non-smoker, FEV1; forced expiratory volume in one second. FVC; forced vital capacity. PC20; the concentration of methacholine required to decrease the FEV1 by 20%, BMI; body mass index. Steroid N; steroid no treatment, Steroid Y: steroid treatment for exacerbation. *p< 0.05 compared with control subjects. # p<0.05 compared with Steriod-N asthma.

**Figure 1:**
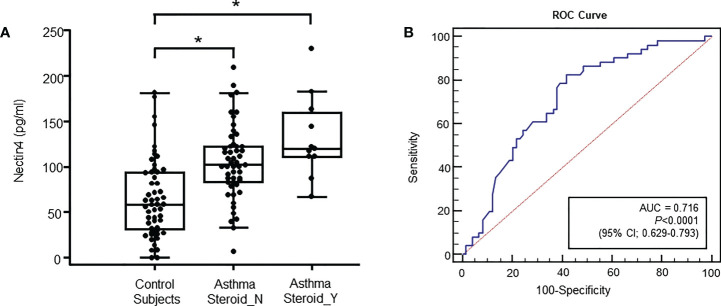
Specimens were obtained from control subjects (n = 60), subjects with non-steroid-treated asthma (n = 51), and subjects with steroid-treated asthma (n = 11) **(A)** Plasma Nectin4 levels in control subjects and asthma patients. *p<0.05, control vs. asthma patients. **(B)** Receiver operating characteristic (ROC) curves of the plasma Nectin4 levels. Nectin4 protein concentrations in asthma patients and controls (Area under the curve (AUC) =0.760, 95% CI 0.673-0.847, p<0.001). The diagonal line represents a hypothetical curve corresponding to a test affording no discriminatory power.

Plasma Nectin4 levels were significantly higher in the latter (68.11 ± 46.38 pg/ml vs. 105.16 ± 40.75 pg/ml or 132.79 ± 45.87 pg/ml p<0.001, **Figure 1A**). During exacerbation, Nectin4 levels tended to be higher in patients with than without steroid medication.

**Figure 2:**
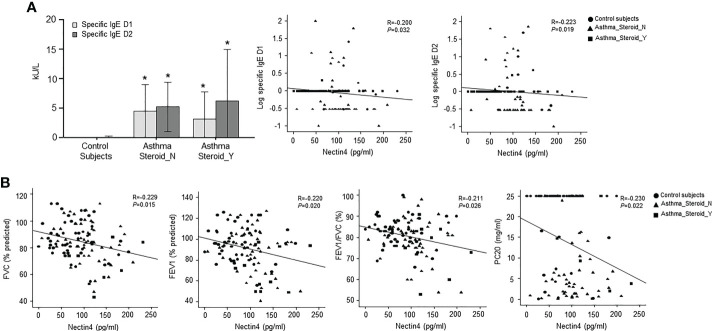
Relationship between plasma Nectin4 and clinical variables in asthma patients. **(A)** Specific IgE D1 or specific IgE D2 level for control subjects, steroid not treated group, and steroid treatment group for exacerbation. *p<0.05, control vs. asthma patients. **(B)** Correlation of plasma Nectin4 and clinical variables including FVC% predicted, FEV1% predicted, FEV1/FVC%, and PC20. * p<0.05, Spearman’s rank test.

The Nectin4 ROC curves for asthma patients and controls are shown in **Figure 1B** (AUC=0.716, p<0.001). A Nectin4 cutoff of 72.54 pg/mL distinguished between asthma patients and controls with 70.25% accuracy, 82.4% sensitivity, and 58.1% specificity (**Figure 1B**). Additionally, the plasma Nectin4 level correlated with specific IgE to *Dermatophagoides pteronyssinus* (D1) and *Dermatophagoides farinae* (D2) (**Figure 2A**), and with FVC% pred. (r  = - 0.229, p = 0.015), FEV1% pred. (r  = - 0.220, p  =  0.020), FEV1/FVC% (r  = - 0.211, p  =  0.026), and PC20 (r  =-  0.230, p = 0.022) (**Figure 2B**).

### *Der* p1 alters Nectin4/Afadin and Src/Rac1 pathways in NHBE cells

**Figure 3:**
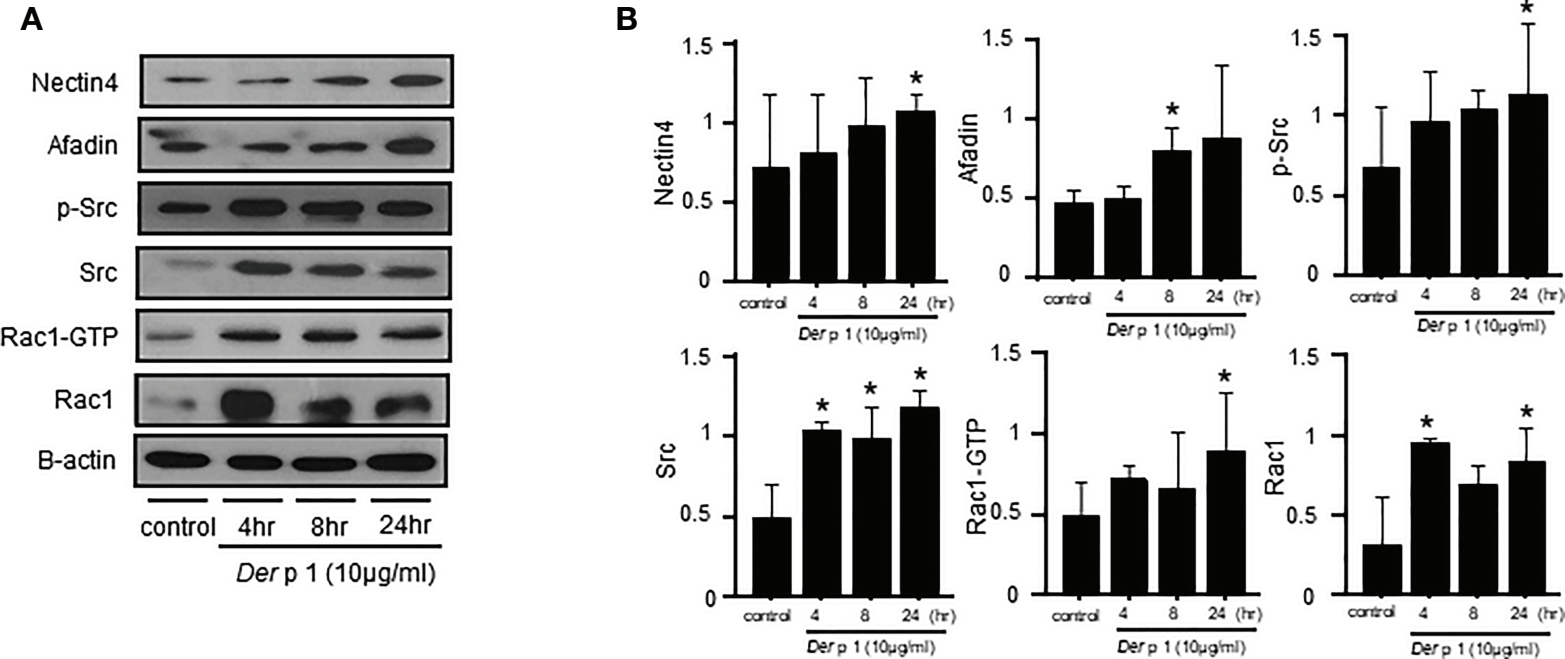
Time courses of *Dermatophagoides pteronyssinus* 1 (*Der* p1) allergen-induced Nectin4/Afadin and Src/Rac1 pathway expression. **(A)** Nectin4/Afadin and Src/Rac1 pathway expression on western blots. **(B)** Densitometry of the bands obtained on three western blots. The values were normalized to those of β-actin and are expressed as the mean ± SEM. The results are representative of at least 3 independent experiments. *p<0.05 compared to the control.

The mechanism by which *Der* p1 alters Nectin4 was investigated by exposing NHBE cells to the allergen, and then measuring Nectin4/Afadin and Src/Rac1 protein levels (**Figure 3**). Both were increased in NHBE cells within 4-, 8-, and 24-h exposure to *Der* p1 (p < 0.05, **Figures 3A, B**).

### Nectin4 knockdown alters Afadin and Src/Rac1 pathway expression in *Der* p1-treated NHBE cells

**Figure 4:**
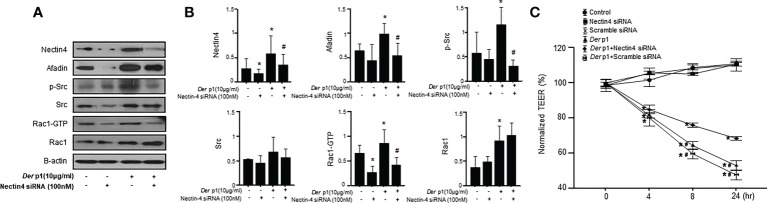
The knockdown of Nectin4 alters the expression of Afadin and the Src/Rac1 pathway. **(A)** Nectin4 knockdown alters the expression of Afadin and the Src/Rac1 pathway in NHBE cells treated with *Der* p1. **(B)** The values were normalized to those of β-actin and are expressed as the means ± SDs. **(C)** Time-dependent increases in TEER in NHBE cells treated with *Der* p1 and decreases in NHBE cells treated with *Der* p1 and Nectin4 siRNA. The results are representative of at least 3 independent experiments. *p<0.05 compared to the control.

The cellular suppression of Nectin4 associated with siRNA transfection reduced Afadin, p-Src, and Rac1-GTP, but not Src or Rac1, expression (**Figures 4A, B**). Changes in the levels of Nectin4/Afadin and Src/Rac1 pathway proteins in NHBE cells treated with both *Der* p1 and siRNA were also observed. The effects of Nectin4 on the AJ permeability of NHBE cells were examined in a Transwell assay, which revealed a decrease in TEER in NHBE cells at 4, 8, and 24 h after *Der* p1 exposure, and an increase 8 and 24 h after Nectin4 siRNA treatment (**Figure 4C**).

### Nectin4 contributes to the development of OVA-induced asthma

**Figure 5:**
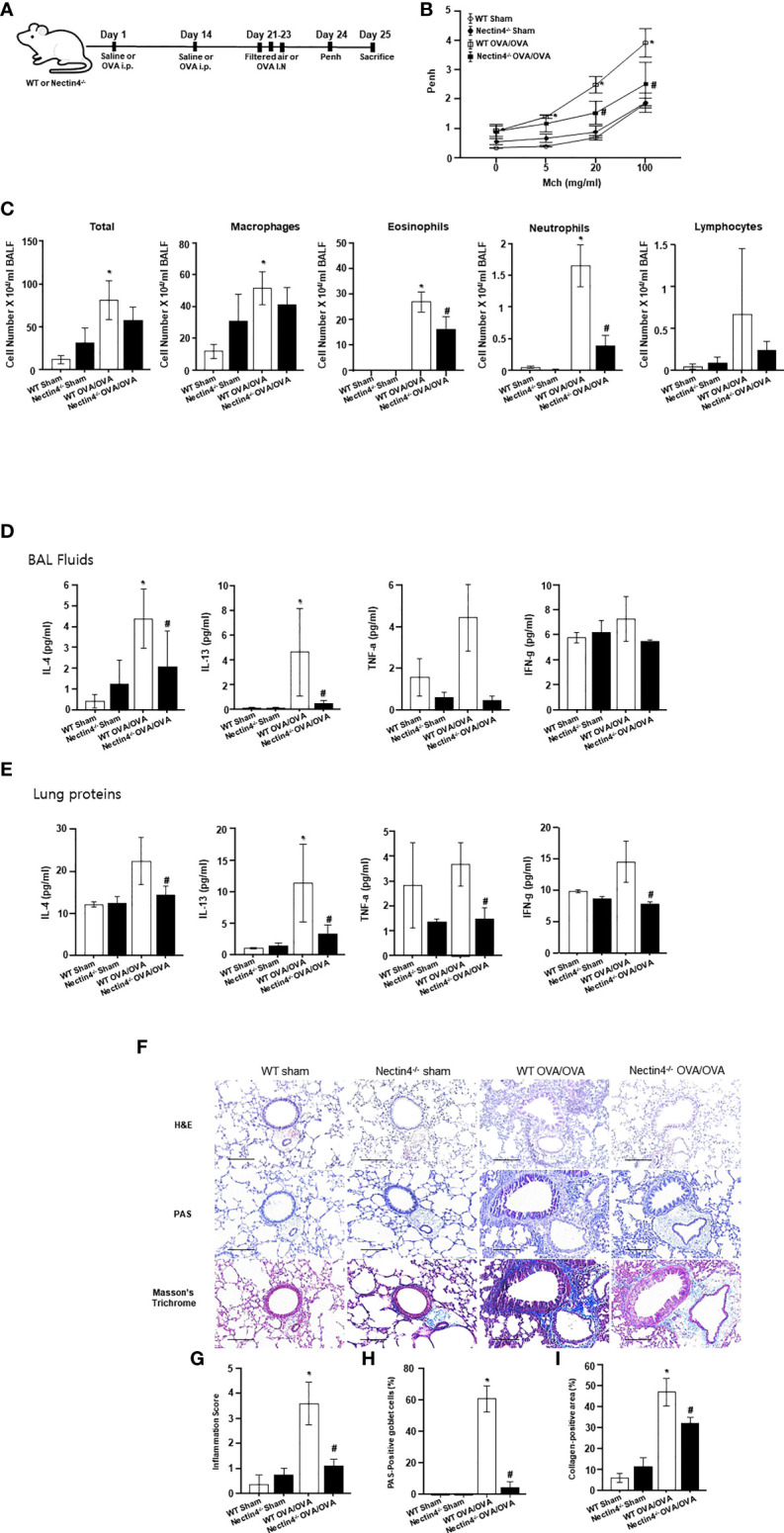
Absence of Nectin4 prevents development of OVA-induced asthma. **(A)** Scheme representing the acute ovalbumin-dependent asthma model of WT and Nectin4^-/-^ mice. **(B)** Airway hyperresponsiveness in response to methacholine (Mch) (n = 6-10 mice per group). **(C)** Inflammatory cells in BAL fluid. **(D, E)** Levels of IL-4, TNF-a, and IFN-g in BAL fluids and Lung proteins (n = 6-10 mice per group). **(F)** Representative images of H&E, PAS, Masson’s trichrome-stained, paraffin-embedded lung sections of asthmatic mice (scale bar = 50 μm). **(G-I)**. Bar graphs summarize means ± SEMs of histological scoring of inflammation grading based on H&E staining, the number of goblet cells based on PAS staining and of fibrosis based on trichrome staining. * p<0.05 vs. WT sham. #p<0.05 vs. WT OVA/OVA.

To determine whether Nectin4 deletion affects features of OVA-induced asthma, Nectin4^−/−^ mice and wild-type (WT) control littermates were sensitized on days 0 and 14 with OVA, and then challenged on days 21 to 23 with OVA or saline (**Figure 5A**). As expected from previous work using the OVA-induced asthma model, AHR expression in response to increasing doses of methacholine increased in WT OVA/OVA mice compared to WT sham mice (**Figure 5B**). Nectin4^−/−^ mice sensitized and challenged with OVA (Nectin4^−/−^ OVA/OVA) had a lower AHR than their WT counterparts when treated with higher doses of methacholine (**Figure 5B**). The numbers of total cells, macrophages, eosinophils, neutrophils, and lymphocytes in BAL fluid were higher in WT OVA/OVA than WT mice (WT sham) (**Figure 5C**), and much lower in Nectin4^−/−^ OVA/OVA than WT OVA/OVA mice (**Figure 5C**). Type 2 cytokine expression (IL-4 and IL-13) in BAL fluid was higher in WT OVA/OVA mice than in WT sham mice (**Figure 5D**). In BAL fluid of Nectin4^−/−^ OVA/OVA mice, and the amounts of IL-4, IL-13 and Transwell Th1 and Th2 cytokines were significantly lower than in WT OVA/OVA mice (**Figures 5D, E**). Histologic analysis showed that WT OVA/OVA mice had mucosal gland hyperplasia and numerous inflammatory cell and collagen infiltrates in peribronchial areas (**Figure 5F**). The lung inflammation score, which measures goblet cells and collagen-positive areas, was 40% lower in Nectin4^−/−^ OVA/OVA mice than WT OVA/OVA mice (**Figures 5G–I**).

### Nectin4 controls Afadin expression and activation of the Src/Rac1 pathway in OVA-induced asthma

**Figure 6:**
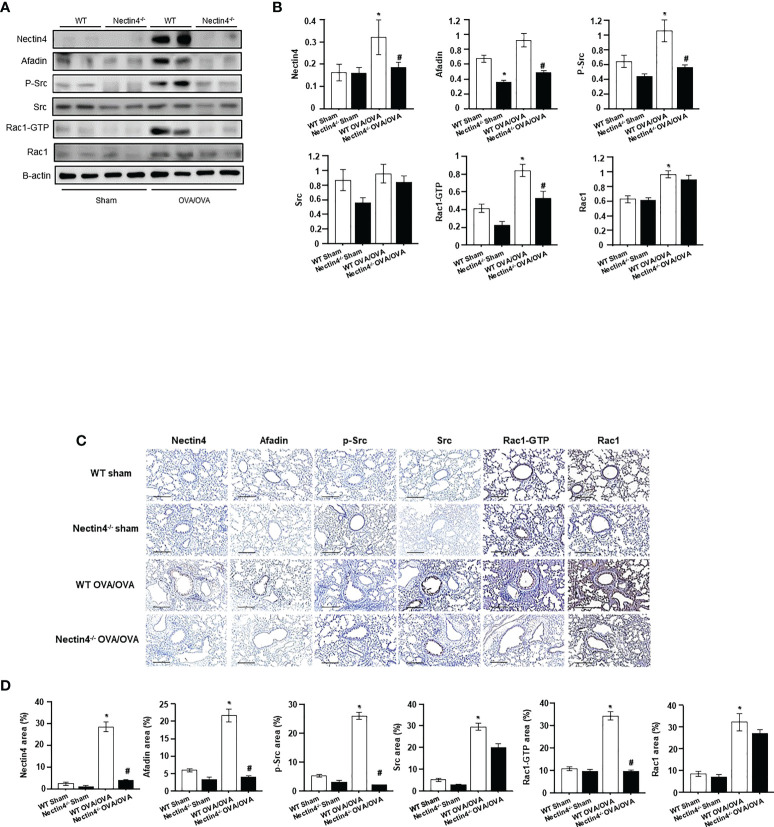
Absence of Nectin4 controls the expression of Afadin and the Src/Rac1 pathway. **(A)** Expression of the Nectin4/Afadin and the Src/Rac1 pathway in ovalbumin-induced asthma model of WT and Nectin4^-/-^ mice. **(B)** Densitometry of the bands obtained on three different individuals western blots. The values were normalized to those of β-actin and are expressed as the mean ± SEMs (n = 6-10 mice/group). **(C)** The lung tissues of OVA-sensitized/challenged mice were analyzed for Nectin4/Afadin and Src/Rac1 pathway expression by immunohistochemical staining (scale bar = 50 μm). **(D)** Quantification of percentage of Nectin4/Afadin and Src/Rac1 pathway protein-positive areas. All quantitative analyses were performed using ImageJ software. The results are representative of at least 3 independent experiments. * p<0.05 vs. WT sham. #p<0.05 vs. WT OVA/OVA.

Total Nectin4/Afadin and Src/Rac1 pathway protein levels in the lung were higher in WT OVA/OVA than WT sham mice (**Figures 6A, B**). Immunohistochemical staining revealed increased Nectin4/Afadin and Src/Rac1 protein levels in the mononuclear inflammatory cells and epithelial cells of WT OVA/OVA mice (**Figures 6C, D**). However, both the expression of Nectin4/Afadin and the levels of the activated form of Src/Rac1 (p-Src/Rac1-GTP) were decreased in Nectin4-/- OVA/OVA mice. No significant differences in Src and Rac1 expression were detected between WT OVA/OVA and Nectin4-/- OVA/OVA mice (**Figure 6**).

### Nectin4 interacts with Rac1-GTP in OVA-induced asthma

**Figure 7:**
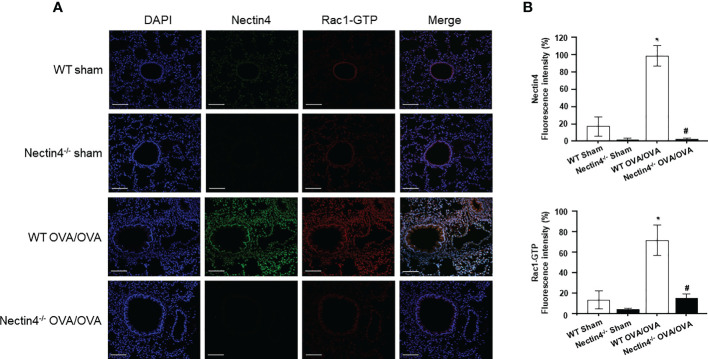
Double-staining immunofluorescence of Nectin4 (Green) and Rac1-GTP (Red) in ovalbumin-induced asthma model of WT and Nectin4^-/-^ mice lung. **(A)** Representative immunofluorescence images of Nectin4 and Rac1-GTP in lung of the mice (scale bar = 100 μm). **(B)** Quantitation of the fluorescence intensity of Nectin4 and Rac1-GTP. * p<0.05 vs. WT sham. #p<0.05 vs. WT OVA/OVA.

To confirm that the activated form of Rac1, Rac1-GTP, was responsible for the airway inflammation seen in Nectin4–/– mice, double-staining immunofluorescence of Nectin4 and Rac1-GTP was performed in lung tissue from mice with OVA-induced asthma. The results showed a lower level of Rac1-GTP expression in Nectin4^–/–^ OVA/OVA than WT OVA/OVA mice (**Figure 7**).

## Discussion

In this study, we explored the role of Nectin4 and its participation in Src/Rac1 signaling in asthma. Nectin4 was shown to be highly expressed in the lung tissue of asthmatic mice, with increased levels also measured in the blood of patients with asthma. These results suggested that Nectin4 is a biomarker of asthma. The pulmonary airway epithelium is a critical external interface but it is often exposed to harmful aerosols and pathogens (20). The proximal bronchial epithelium comprises columnar ciliated cells and mucus-secreting goblet cells supported by basal cells. Together, they form a selective permeability barrier to control fluid loss, the entry of pathogens, and inappropriate immune reactions in the subepithelial lung mucosa (20).

The Nectin family comprises five transmembrane glycoproteins (PVR/CD155, Nectin-1/CD111, Nectin-2/CD112, Nectin-3, and Nectin4), all members of the immunoglobulin superfamily. Nectin proteins are both homophilic and heterophilic cell adhesion molecules (21, 22). Soluble Nectin4 was detected both in the supernatant of breast tumor cell lines and in 51% of the sera of patients with metastatic breast carcinoma (14). Among the mechanisms accounting for Nectin4 release in cell supernatants are cell degradation, proteolytic processes, and alternative splicing (14). In our study, plasma Nectin4 levels were higher in asthmatic patients than in control subjects. The relationship of Nectin4 levels to lung function and airway responsiveness suggested the secretion of a circulating form of Nectin4 in patients with asthma, and thus its utility both as a blood marker for the diagnosis of asthma and monitoring of disease progression. A previous study showed that the overexpression of Nectin4 alters the epithelial architecture of NHBE cells, leukocyte transmigration, and diapedesis (14). These findings, and those of the present study, suggest that soluble Nectin4 is involved in the inflammatory process of asthma.

As a candidate host exit receptor, Nectin4 (poliovirus-receptor-like-4) interacts with high affinity with viral attachment proteins through its membrane-distal domain (23). Nectin4 sustains measles virus entry and the non-cytopathic lateral spread of the virus in well-differentiated primary human airway epithelial sheets infected basolaterally (23). The downregulation of Nectin4 in infected epithelial cells, including those of the macaque trachea, has been reported (23). In our study, Nectin4 protein expression was increased in the blood of asthma patients, and in the lung tissue in a mouse model of asthma. The increased levels of Nectin4 in NHBE cells treated with *Der* p1 may allow Nectin4 to enter the airways or penetrate their epithelial barriers. Nectins initiate cell–cell adhesion by their *trans*-interactions and, *via* Afadin, an actin filament-binding protein that connects Nectins to the cytoskeleton (24), to recruit other proteins to establish adherens and then tight junctions (25). The four terminal amino acids of Nectin4 bind Afadin, which tethers Nectin4 to F-actin (26, 27). In accordance with previous studies (24–27), in this study Nectin4/Afadin was expressed in the inflamed airway tissue in a mouse model of asthma. This finding suggests that the cytoskeletal connection of Nectin4 and Afadin facilitates allergen entry and thus contributes to airway inflammation.

The Rho family GTPase Rac1 is a key regulator of many cellular functions, such as cytoskeletal reorganization and cell growth (28–30). It has also been implicated in antibacterial host defenses, including leukocyte chemotaxis (29), pathogen phagocytosis (31, 32), ROS production (33), and the regulation of TLRs and NOD2 (34–37). The cadherin complex, including β-catenin, Rho, and Rac, regulate the assembly and disassembly of cell junctions (38, 39). In our study, Src and Rac1 protein expression were increased in the lung tissue of a mouse model of asthma, and in NHBE cells treated with *Der* p1, indicating that Src/Rac1 signaling is involved in Nectin4 expression. In the allergen sensitization mice model, and in NHBE cells treated with *Der* p1, the allergen activated Rac1 (Rac1-GTP), leading to an increase in Nectin4 expression, airway inflammation, and AHR. Whether the Rac1-dependent regulation of cell barriers plays an important role in airway inflammation and thus in asthma remains to be elucidated in further studies examining the links between the two proteins. In NHBE cells exposed to *Der* p1 both Nectin4 and Afadin expression were induced *via* the activation of Src/Rac1 signaling pathways. A similar mechanism can be proposed in human airway epithelial cells. This hypothesis is supported by the observed reductions in Afadin stimulation and the Src/Rac1 cascade pathway following *Der* p1 exposure in cells pretreated with a Nectin4 small interfering RNA (siRNA).

Previous studies reported that the knockdown of Nectin4 alters cell proliferation, expression of cell-survival-related molecules, and regulation of angiogenesis (40, 41). Nectin4 knockdown also decreased Akt and Src phosphorylation and suppressed angiosarcoma in an *in vivo* xenograft model (41, 42). Little is known about how Nectin4 regulates target protein levels and apoptosis, especially in asthma. Our study examined the involvement of Nectin4 in asthma by assessing airway responsiveness and inflammation in Nectin4^−/−^ mice. Decreases in airway responsiveness and inflammation as well as in type 1 and 2 cytokines, such as IL-4, IL-13, TNF-α, and IFN-γ, were determined in the BAL fluid of Nectin4^−/−^ mice. Using mouse lung samples, we showed that, compared to wild type asthmatic mice, the knockdown of Nectin4 alters the expression of both Afadin and the activated form of Src and Rac1. This result indicated the involvement of Nectin4 in asthma, by regulating Src and Rac1.

In conclusion, our study demonstrated the potential role of Nectin4 in asthma. The detection of a circulating form of Nectin4 in the blood of patients with asthma suggests the involvement of Nectin4 in the initiation of airway inflammation *via* a mechanism that includes the Src/Rac1 cascade pathway. Nectin4 may therefore be a biomarker and potential therapeutic target in asthma.

## Data availability statement

The original contributions presented in the study are included in the article/supplementary material. Further inquiries can be directed to the corresponding author.

## Ethics statement

The studies involving human participants were reviewed and approved by Soonchunhyang University Bucheon Hospital Institutional Review Board. The patients/participants provided their written informed consent to participate in this study. The animal study was reviewed and approved by Institutional Animal Care and Use Committee of the Soonchunhyang University Bucheon Hospital.

## Author contributions

Conceptualization: A-SJ, P-HL. Methodology: P-HL, SC, MA, DH. Investigation: A-SJ, P-HL, SP, AB. Visualization: P-HL, SC, MA, DH. Funding acquisition: A-SJ. Writing – original draft: A-SJ, P-HL. Writing – review & editing: A-SJ, P-HL, SP, AB. All authors read and approved the final manuscript. All authors contributed to the article and approved the submitted version.

## Funding

This research was supported by Basic Science Research Program through the National Research Foundation of Korea (NRF) funded by the Ministry of Science and ICT (NRF-2020R1A2C1006506) and Soonchunhyang University.

## Conflict of interest

The authors declare that the research was conducted in the absence of any commercial or financial relationships that could be construed as a potential conflict of interest.

## Publisher’s note

All claims expressed in this article are solely those of the authors and do not necessarily represent those of their affiliated organizations, or those of the publisher, the editors and the reviewers. Any product that may be evaluated in this article, or claim that may be made by its manufacturer, is not guaranteed or endorsed by the publisher.
